# Glycosome biogenesis in trypanosomes and the de novo dilemma

**DOI:** 10.1371/journal.pntd.0005333

**Published:** 2017-04-20

**Authors:** Sarah Bauer, Meredith T. Morris

**Affiliations:** Eukaryotic Pathogens Innovation Center, Department of Genetics and Biochemistry, Clemson University, Clemson, South Carolina, United States of America; Yale School of Public Health, UNITED STATES

## Abstract

Trypanosomatid parasites, including *Trypanosoma* and *Leishmania*, are the causative agents of lethal diseases threatening millions of people around the world. These organisms compartmentalize glycolysis in essential, specialized peroxisomes called glycosomes. Peroxisome proliferation can occur through growth and division of existing organelles and de novo biogenesis from the endoplasmic reticulum. The level that each pathway contributes is debated. Current evidence supports the concerted contribution of both mechanisms in an equilibrium that can vary depending on environmental conditions and metabolic requirements of the cell. Homologs of a number of peroxins, the proteins involved in peroxisome biogenesis and matrix protein import, have been identified in *T*. *brucei*. Based on these findings, it is widely accepted that glycosomes proliferate through growth and division of existing organelles; however, to our knowledge, a de novo mechanism of biogenesis has not been directly demonstrated. Here, we review recent findings that provide support for the existence of an endoplasmic reticulum (ER)-derived de novo pathway of glycosome biogenesis in *T*. *brucei*. Two studies recently identified PEX13.1, a peroxin involved in matrix protein import, in the ER of procyclic form *T*. *brucei*. In other eukaryotes, peroxins including PEX13 have been found in the ER of cells undergoing de novo biogenesis of peroxisomes. In addition, PEX16 and PEX19 have been characterized in *T*. *brucei*, both of which are important for de novo biogenesis in other eukaryotes. Because glycosomes are rapidly remodeled via autophagy during life cycle differentiation, de novo biogenesis could provide a method of restoring glycosome populations following turnover. Together, the findings we summarize provide support for the hypothesis that glycosome proliferation occurs through growth and division of pre-existing organelles and de novo biogenesis of new organelles from the ER and that the level each mechanism contributes is influenced by glucose availability.

## Glycosomes enable parasite survival in multiple environments

*T*. *brucei* is the protozoan parasite responsible for human African trypanosomiasis (HAT), a lethal disease affecting over 15,000 people in sub-Saharan Africa [1], and nagana, a wasting disease in cattle [2]. *T*. *brucei* belongs to Kinetoplastea, a class of protozoan parasites named for a mass of mitochondrial DNA situated at the base of the flagellum in a single, intricate mitochondrion called the kinetoplast [2,3]. This class also includes *T*. *cruzi*, the causative agent of American trypanosomiasis, and the genus *Leishmania*, encompassing a number of species responsible for leishmaniasis [2–5]. Combined, these diseases threaten millions of people and have serious social and economic impacts [1]. Due to the high cost of treatment, harsh side effects, and rise in resistance to currently available drugs, identifying new drug targets is crucial [1,3].

*T*. *brucei* parasites are transmitted by tsetse flies. Bloodstream form (BSF) parasites dwell in the bloodstream and lymphatic system of their mammalian hosts [6]. Recently, parasites were also identified in skin and adipose tissue [7]. When a tsetse fly takes a blood meal, parasites differentiate into procyclic forms (PFs) in the midgut of the fly. PFs then migrate to the salivary glands, differentiate into metacyclic forms, and can be transmitted to another mammalian host [2,8,9]. *T*. *brucei* encounters multiple environments with different available nutrients. In the mammalian bloodstream, glucose is abundant, being maintained at ~5 mM, and BSF parasites rely solely on glycolysis for the generation of ATP. In the tsetse fly midgut, glucose levels fall to undetectable levels within 15 minutes of a bloodmeal [9].

Unlike other eukaryotes in which glycolysis is a cytosolic process, *T*. *brucei* and other kinetoplastids compartmentalize enzymes involved in glycolysis in organelles called glycosomes [5,10,11]. These organelles are evolutionarily related to peroxisomes and harbor many proteins homologous to peroxisome proteins, which are involved in import and biogenesis in higher eukaryotes. These are called peroxins [3,5].

Glycosomes are essential to parasite survival, likely due to compartmentalization of key metabolic enzymes. For example, the glycolytic enzymes hexokinase and phosphofructokinase are not inhibited by their products, and compartmentalization of glycolysis likely prevents these enzymes from depleting ATP within the cell [11,12]. Disruption of glycosome biogenesis or glycosome protein import results in mislocalization of these enzymes to the cytoplasm and cell death [13,14].

Glycosome composition changes during parasite lifecycle. In BSF parasites, ATP is generated exclusively through glycolysis, and ~90% of glycosomal protein content is involved in glycolysis [5,15]. In contrast, PF parasites generate ATP via glycolysis when glucose is present but switch to amino acid metabolism when it is absent [10,16]. In this stage, less than 50% of the glycosome protein repertoire is involved in glycolysis [5,15]. Unlike BSFs, PFs have a fully functional mitochondrion and proteins involved in the generation of ATP, including the tricarboxylic acid (TCA) cycle, electron transport chain, and proline and threonine metabolism that are located in the mitochondrion are upregulated [17].

The essential nature of glycosomes and the uniqueness of the organelles make them ideal targets for drug development [18]. While much about glycosomal matrix protein import and organelle function in *T*. *brucei* has been resolved, understanding of glycosome biogenesis is lacking. Specifically, it is unclear the extent to which glycosome proliferation occurs through the multiplication of existing organelles or biogenesis from the ER. Here, we discuss our current understanding of glycosome biogenesis and summarize data supporting the existence of a de novo mechanism of glycosome biogenesis.

## Glycosomes are related to peroxisomes, which proliferate by both fission of existing organelles and de novo biogenesis of new organelles from the ER

Glycosomes are considered specialized peroxisomes because they share similar structure, function, and homologous peroxins involved in protein import and biogenesis. Both organelles are membrane-bounded microbodies with electron-dense matrices. They lack DNA and import proteins post-translationally [11,19,20]. Peroxisomes are diverse organelles, and activities vary with organism, cell type, and environment [21]. While glycosomes share a number of pathways with peroxisomes, they are unique in that they harbor enzymes involved in glycolysis [3,11,20,22].

Glycosomes harbor homologs to yeast, mammal, and plant peroxin proteins that participate in matrix protein import, membrane protein insertion, and organelle fission [3,5]. At least 13 peroxin homologs have been identified in kinetoplastids, and although the overall sequence identity of these peroxins range between 15% and 35%, when compared to homologs in other organisms, their activities are well conserved [5]. Like peroxisomes, glycosomal matrix proteins are targeted for import by the peroxisomal targeting signal (PTS) PTS1 or PTS2. In eukaryotes, including kinetoplastids, PTS1s are three amino acid residues ((S/A/C)-(K/R/H)-L) located on the C-terminus of glycosomal proteins, and the PTS2 consists of a longer sequence ((R/K)(L/V/I)X_5_-(Q/H)(L/A)) located at N-terminus of glycosomal matrix proteins. These signals are recognized by cytosolic receptor peroxins (PEX5 and PEX7 for PTS1 and PTS2, respectively). PEX5 and PEX7 associate with a docking complex in the glycosomal membrane that contains PEX13 and PEX14, where the cytosolic receptor and cargo are translocated into the glycosomal matrix [3,5]. While recycling of PEX7 is not well understood, PEX5 is mono-ubiquitinated by the ubiquitin-conjugating enzyme, PEX4, and recycled back into the cytoplasm or polyubiquitinated and targeted for degradation [5,21].

Peroxins also regulate the abundance and size of peroxisomes and glycosomes. Growth and division of peroxisomes is regulated by the integral membrane peroxin, PEX11, which directs elongation of peroxisomes that then undergo constriction by dynamin-like GTPases [21,23]. Silencing of PEX11 in *Saccharomyces cerevisiae* blocks fission, resulting in fewer, larger peroxisomes [23,24]. In *T*. *brucei*, TbPEX11 silencing results in fewer, larger glycosomes, and overexpression yielded abnormal glycosomes. Additionally, TbPex11 expressed in CV-1 monkey cells colocalized with peroxisome proteins. These findings, and the observation that TbPEX11 complemented functionally *pex11*Δ knockout yeast, indicate it is functionally related to Pex11 in higher eukaryotes [25].

Mechanisms of de novo biogenesis have been characterized in yeasts and mammalian cells. PEX3, PEX16, and PEX19 are hallmark peroxins for this pathway. PEX19 is a cytosolic receptor that recognizes membrane peroxisomal targeting signals (mPTSs) on peroxisomal membrane proteins (PMPs). Like matrix proteins, most PMPs are synthesized on free ribosomes in the cytosol. The PEX19–PMP complex docks with PEX3, an integral peroxisomal membrane peroxin involved in the insertion of PMPs [3,21,23,26]. Although the function of PEX16 differs slightly among different species, in mammalian cells, it recruits PEX3 and other PMPS to the ER [26,27]. Mutation of PEX3 or PEX19 in yeast and PEX3, PEX16, or PEX19 in mammalian cells results in cells without peroxisomes, which are restored upon complementation with functional gene copies, demonstrating the importance of these peroxins in de novo peroxisome biogenesis [28–40].

Two mechanisms of ER-dependent peroxisome proliferation have been proposed (reviewed in [21,26]). In the heterotypic fusion model, yeast PEX3 forms preperoxisomal domains within the ER. Other PMPs, including docking complex peroxins (PEX13 and PEX14) and the really interesting novel gene (RING) finger peroxins (PEX2, PEX10, and PEX12) involved in PEX5 receptor recycling, are trafficked to the ER. Preperoxisomal vesicles (ppVs) containing either docking complex PMPs or RING finger proteins bud from the ER independently. These import-incompetent ppVs, each containing a portion of the import machinery, can fuse and form import-competent peroxisomes [41].

In the second model, vesicle maturation, PEX3 localizes to the ER in a PEX16-dependent process. These ppVs, containing PEX3 and PEX16, bud from the ER, import PMPs, and mature into organelles that support protein import [42–44]. These ppVs can fuse with currently existing peroxisomes, contributing new lipids and PMPs to the growth and division mechanism of biogenesis [45,46].

While our understanding of protein targeting and import into glycosomes has grown, our understanding of how these organelles are formed and proliferate (i.e., glycosome biogenesis) is lacking. Several issues complicate the study of de novo biogenesis in *T*. *brucei*. Although TbPEX16 and TbPEX19 have been characterized [47,48], the key peroxin in de novo biogenesis of peroxisomes in other eukaryotes, PEX3, has not been identified in any kinetoplastid genome. With the low sequence identity between homologous peroxins in different species, this is not unexpected [5], and we are using biochemical approaches to identify a PEX3 homolog in *T*. *brucei*. Additionally, studies of de novo biogenesis in other systems rely on complementing peroxisomes in cell lacking these organelles [49]. Such studies are challenging in *T*. *brucei* because glycosomes are essential [18]. Despite these technical challenges, recent findings support the hypothesis that glycosomes can be made de novo in *T*. *brucei*.

## Recent studies suggest glycosomes can proliferate de novo in trypanosomes

In yeast and mammals, several peroxins localize to the ER during de novo biogenesis [21,23,26,49]. In pulse-chase experiments, PEX3 fused to yellow fluorescent protein localized to ER foci before being trafficked to peroxisomes [44]. Other PEXs, including PEX2, PEX3, PEX8, PEX10, PEX11, PEX13, PEX14, PEX15, PEX16, PEX17, PEX30, and PEX31, also localize to ER in areas where ppVs are generated [26,41,42,44,50–54]. Furthermore, in mouse dendritic cells, PEX13 localized to lamellar structures that bud from the ER, giving rise to mature peroxisomes [55].

Two independent studies have found the trypanosome peroxin TbPEX13.1 associated with the ER [56]. Trypanosomes are unique in that they have two essential, nonredundant PEX13s, TbPEX13.1 and TbPEX13.2. While they share low sequence identity (less than 16%), both localize to glycosomes and function in matrix protein import [57,58]. In a recent study, Güther et al. used affinity purification to isolate glycosomes from PF cells expressing GFP-tagged TbPEX13.1. Quantitative proteomics identified ER proteins along with glycosome proteins enriched in the purified fractions [56]. Additionally, our lab found that TbPEX13.1 localized to the ER in PF *T*. *brucei* under low-glucose conditions [59]. Because peroxins localize to the ER in other organisms during de novo biogenesis, it is interesting to speculate that TbPEX13.1 localizes to foci in the ER where new glycosomes or preglycosomal vesicles bud. Resolving how TbPEX13.1 is localized to different compartments will provide insight into possible mechanisms that regulate glycosome biogenesis. Additionally, it will be important to determine if TbPEX13.2 or other peroxins exhibit such localization patterns. These studies are ongoing in our laboratory.

PEX16 plays a pivotal role in de novo biogenesis of peroxisomes in some yeast and mammalian cells [21,23,26,27,49]. In *Yarrowia lipolytica*, PEX16 negatively regulates peroxisome fission [60]. *Y*. *lipolytica* PEX16s are N-glycosylated in the ER, and mutation to ER exit machinery blocks ER exit [27,50]. In human cells, PEX16 ER exit depends on the protein SEC16B [61,62]. TbPEX16 was recently characterized in BSF and PF *T*. *brucei*. TbPEX16 silencing resulted in a decrease in glycosome number. Remaining glycosomes were no longer distributed throughout the cell but sequestered to the posterior half of the parasite [48]. *T*. *brucei* has a single SEC16, which is homologous to human SEC16B [48,63]. The TbSEC16 ER exit site is located between the kinetoplast and nucleus [63] in the anterior end of the trypanosome, opposite of where glycosomes are sequestered in cells lacking TbPEX16 [48]. The authors propose that TbPEX16 is targeted to the ER and that silencing interferes with the budding of glycosomes through the TbSEC16 ER exit site, yielding a decreased number of organelles in this area of the cell [48].

PEX19 also plays an important role in the de novo biogenesis of peroxisomes. Silencing PEX19 in *S*. *cerevisiae* and *Hansenula polymorpha* resulted in cells lacking peroxisomes, while complementation with functional variants of PEX19 restored peroxisome populations [31,39,40]. Analogous results were observed in mammalian cells [34]. Similarly, TbPEX19 silencing slowed PF parasite growth in media containing glucose and resulted in a decrease in the total number of glycosomes per cell. In the absence of glucose, TbPEX19 silencing had a less dramatic effect on growth, likely because mislocalization of glycosomal matrix proteins is not lethal under those conditions [47]. These results suggest it may be possible to knock out TbPEX19 under low-glucose conditions to generate aglycosomal parasites. In such a system, complementation experiments could demonstrate the existence of an ER-dependent mechanism of glycosome biogenesis if glycosome populations are restored upon reintroduction of TbPEX19.

Peroxisomes are degraded through an autophagic process called pexophagy, which is often triggered by environmental factors [64]. In *S*. *cerevisiae*, fatty acids trigger the multiplication of peroxisomes that are degraded when cells are moved to glucose media [65]. Environmentally induced changes in peroxisome composition also occur in the methylotrophic yeast *H*. *polymorpha*. In these cells, peroxisomes that are induced in methanol are degraded when cells are transferred to glucose [66].

**Fig 1:**
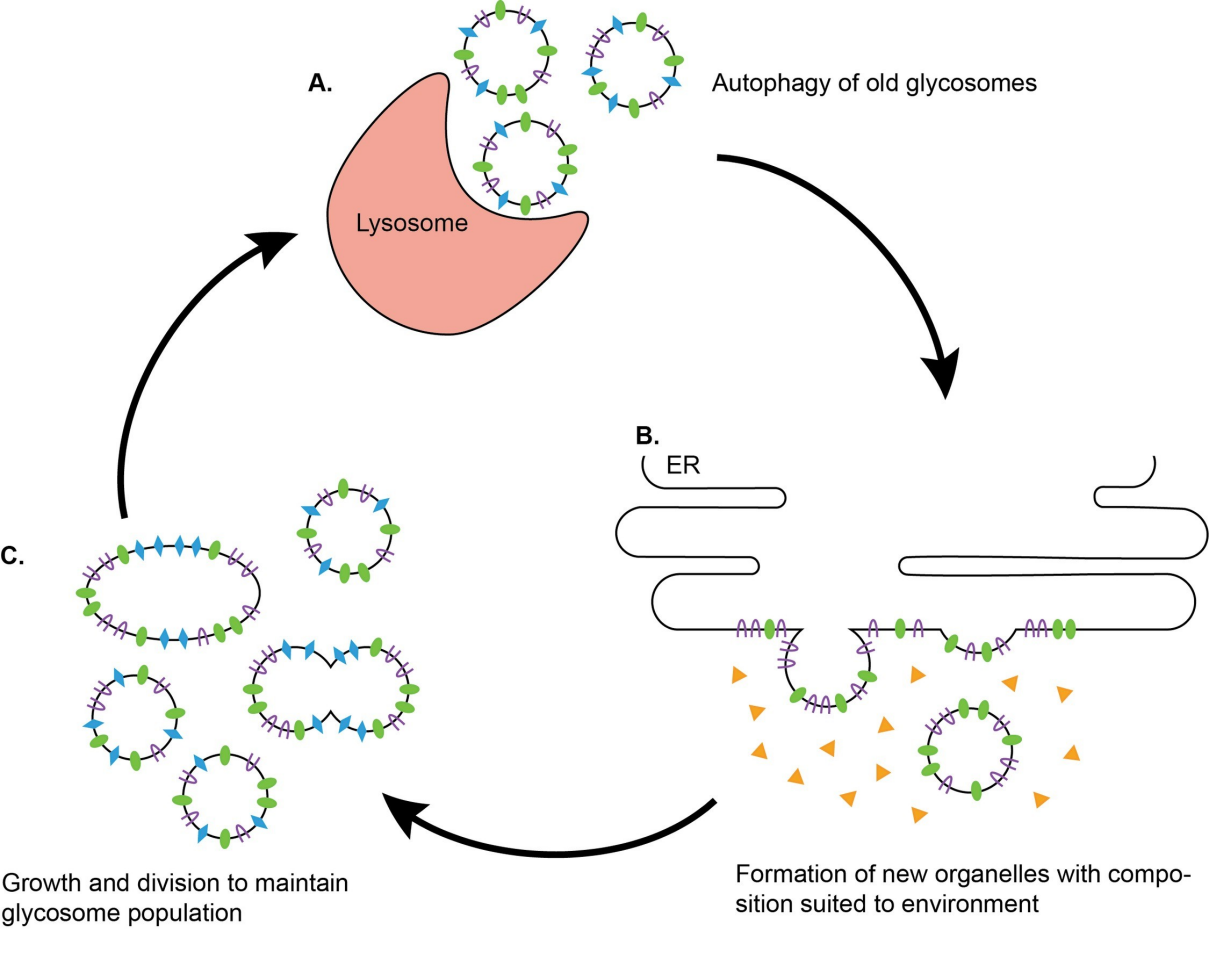
De novo biogenesis may facilitate production of new glycosomes after autophagy of old organelles. (A) Autophagy enables turnover of existing glycosomes [67]. Glucose availability changes through the life cycle of *T*. *brucei*, ranging from undetectable levels to ~5 mM [9]. In response to fluctuations in glucose availability, “old” glycosomes with compositions best suited for prior environmental conditions can be degraded via autophagy. This may explain why glucose-dependent changes in glycosome composition occur in a time frame consistent with autophagy [68]. (B) “New” organelles with protein repertoires better suited to the new levels of glucose in the environment can be generated from the ER [48,49,59,60,68–70]. (C) Once new glycosomes are generated, they can be maintained through fission [26]. This process enables cells to remodel glycosome composition and adapt to changing glucose levels.

*T*. *brucei* parasites encounter multiple environments in which nutrient availability varies. Extracellular glucose levels fluctuate between ~5 mM in the mammalian bloodstream and undetectable levels in the tsetse fly vector [9]. Concomitant with these changes, glycosome composition is altered [22,67–69]. Like peroxisomes, glycosomes associate with lysosomes and are degraded through autophagy during BSF-to-PF differentiation [67]. Glucose fluctuations also influence glycosome composition in PF parasites in a time frame (~3 hr) consistent with autophagy [68,69]. Generation of new glycosomes could provide a way to restore the glycosome population with organelles containing a protein repertoire best suited for the cell’s new environment (Fig 1).

## Glycosomes biogenesis is likely influenced by extracellular glucose levels

De novo peroxisome biogenesis is well established in yeast and mammals but has not been directly demonstrated in kinetoplastids. Because of the similarities in glycosome and peroxisome mechanisms of matrix protein import, PMP insertion, and organelle remodeling, it seems logical that glycosome proliferation could also occur through an ER-dependent process. Localization of peroxins to the ER, analogous phenotypes obtained from silencing key de novo biogenesis peroxins, and similarities in organelle maintenance and turnover provide support that, like peroxisomes, de novo biogenesis of glycosomes occurs in *T*. *brucei*.

There is contradicting evidence regarding the primary mechanism of peroxisome biogenesis in higher eukaryotes. The current consensus is that growth and division of existing organelles and formation of new organelles de novo occur synergistically and that the level each mechanism contributes varies depending on organism, cell type, and environment. Pulse-chase and mating assays in yeast suggest that fission is the primary means of peroxisome multiplication, while the ER serves to supply existing peroxisomes with membrane components [45]. In contrast, the trafficking of PMPs through the ER in wild-type and mutant yeast [70] and the formation of heterotypic vesicles that bud from the ER [41] support a model where de novo biogenesis plays a large role in peroxisome proliferation in yeast. Recent work suggests that both processes function simultaneously [71]. Modeling studies by Mukherji et al. predicted that yeast peroxisomes were produced primarily through fission when cells are grown on oleic acid but switch to de novo biogenesis when the organism is transferred to media containing glucose [26,72]. Recent re-analysis of that work by Crave supports the proposal that, while nutrient conditions influence the mode of proliferation, the changes were less dramatic than originally proposed [73].

**Fig 2:**
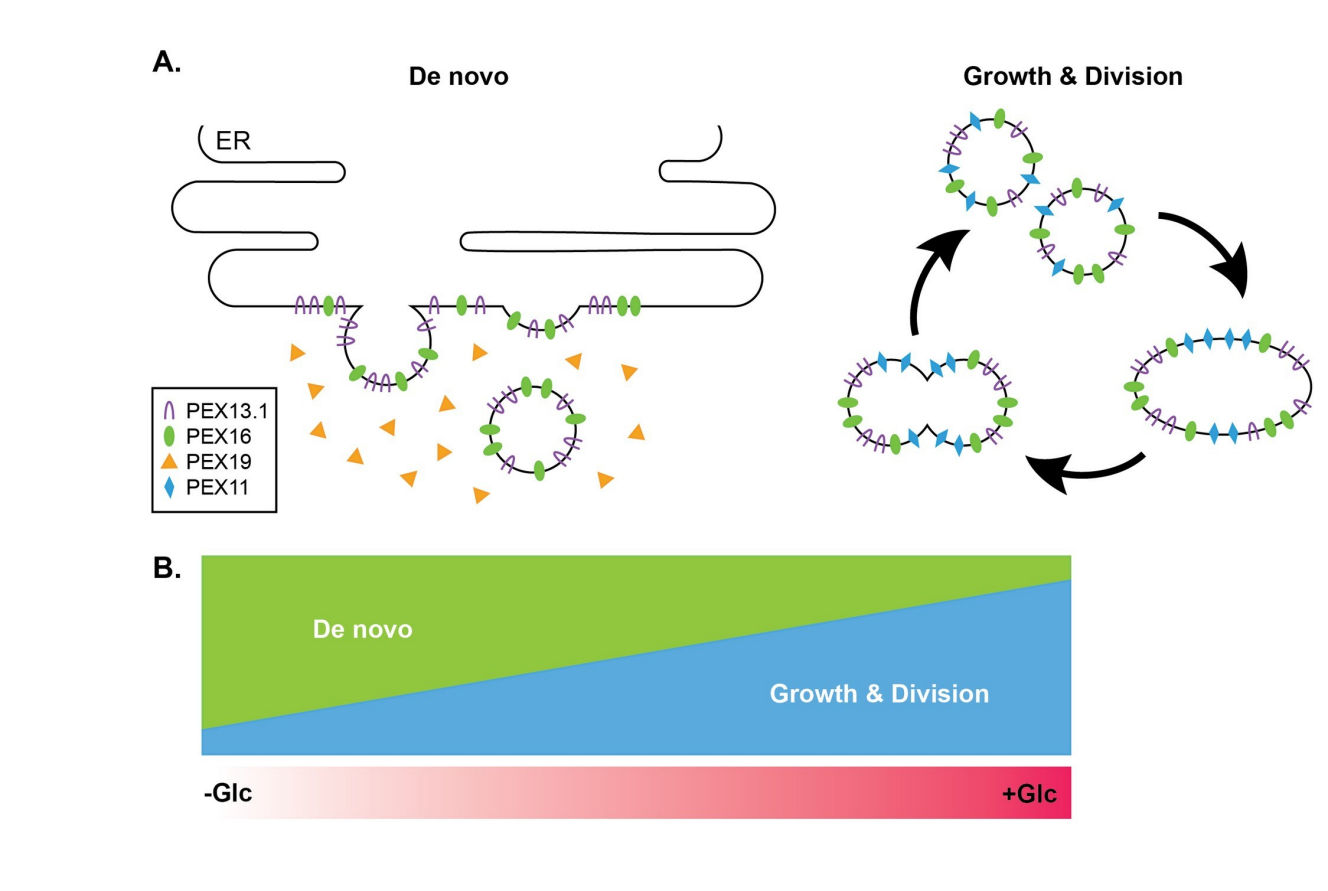
Proposed model for influence of extracellular glucose levels on glycosome biogenesis. (A) When parasites are grown in media containing constant levels of 5 mM glucose, glycosomes proliferate rapidly through TbPEX11-dependent growth and division of pre-existing organelles to facilitate rapid import of glycosome proteins that are toxic if mislocalized. In low-glucose conditions, the slower de novo biogenesis mechanism plays a larger role in glycosome proliferation. Under glucose-poor conditions, the need for rapid import is not essential. In this pathway, TbPEX13.1 and TbPEX16 are first inserted into the ER membrane via TbPEX19 and get incorporated into budding preglycosomal vesicles. Glucose-dependent regulation of glycosome biogenesis could explain why TbPEX13.1 exhibits ER localization under -Glc conditions [59] and silencing of TbPEX16 and TbPEX19 does not result in a more dramatic reduction of glycosome number in +Glc conditions [47,48]. (B) Growth and division and de novo mechanisms of glycosome biogenesis occur synergistically and the level at which each mechanism contributes is dependent on the absolute level of glucose.–Glc (glucose-poor, < 0.05 mM), +Glc (glucose-rich, ~5 mM).

We hypothesize that de novo biogenesis and fission of existing organelles both occur in PF *T*. *brucei* and that environmental triggers, particularly glucose availability, influence the level at which each contributes. We propose that, in high-glucose conditions, glycosomes multiply primarily through the multiplication of existing organelles, while in low-glucose conditions, glycosomes proliferate de novo from the ER (Fig 2).

**Fig 3:**
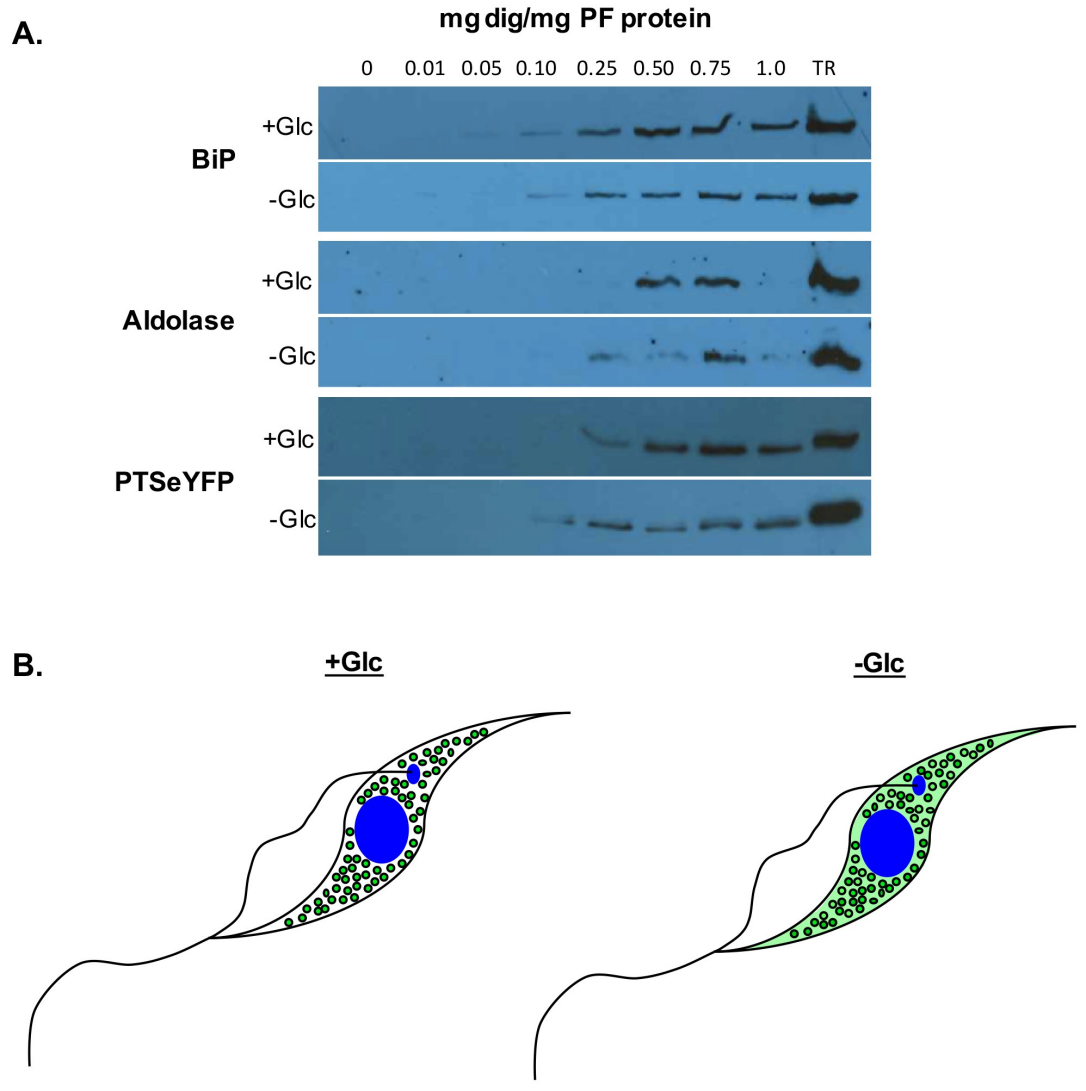
Glycosomal matrix protein import is more tightly regulated when cultured in glucose rich media. (A) Digitonin fractionations of PF cells grown in high glucose (+Glc) and low glucose (-Glc) reveal that glycosomal proteins are mislocalized under low-glucose conditions. Digitonin binds to membrane cholesterol. At lower concentrations, the plasma membrane is solubilized. Higher concentrations are required to dissolve intracellular membranes. Using this protocol, cytoplasmic proteins are released at concentrations below 0.05 mg dig/mg PF protein [74]. Lysates were treated with increasing concentrations of digitonin and intact organelles recovered by centrifugation. Pelleted samples were analyzed by western blotting with antibodies against an ER marker (BiP), a glycosomal matrix protein (aldolase), and a glycosomal reporter protein consisting of a peroxisomal targeting signal (PTS2) fused to an eYFP (PTSeYFP). While BiP is released at 0.10 mg dig/mg PF protein under both conditions, aldolase and PTSeYFP are released at lower concentrations of digitonin under -Glc conditions when compared to +Glc conditions. Aldolase is released at 0.50 mg dig/mg PF protein in +Glc conditions and 0.25 mg dig/mg PF protein in cells grown in -Glc condition. Similarly, PTSeYFP is released at 0.25 mg dig/mg PF protein in cells cultured in +Glc conditions and 0.10 mg dig/mg PF protein under -Glc conditions. The difference in glycosomal protein release and not the control ER resident protein BiP suggests that mislocalized glycosomal proteins can be detected extraglycosomally under low-glucose conditions. (B) Model of glycosome protein mislocalization in low glucose. In high glucose, glycosomal proteins localize exclusively to glycosomes, and mislocalization of any glycosomal proteins (or possibly a particular subset of glycosomal proteins) may be lethal to PF cells. Under low-glucose conditions, mislocalization of glycosomal matrix proteins is more tolerated [12,13], and matrix proteins can be detected in the cytoplasm prior to import. Glycosomal proteins depicted in green.

Generation of peroxisomes through fission of existing organelles is kinetically faster than generation from the ER [21]. We hypothesize that, in glucose-rich media, glycosomes proliferate rapidly by fission, allowing maintenance of metabolic flux while preventing glucose toxicity (Fig 3). Although de novo processes are slower, they may provide a means of generating new glycosomes after the autophagic degradation of old organelles [21]. Degrading “old” glycosomes could yield a temporary source of substrates to satisfy metabolic needs (in the form of glycosome resident proteins) while degrading “old” glycosomal proteins that are unnecessary or detrimental for new metabolic pathways. These new glycosomes may have a composition better suited for growth under glucose-poor conditions.

We hypothesize that under conditions in which glycosomes are generated de novo, PEX13, PEX16, and potentially other membrane peroxins localize to the ER at foci where new glycosomes bud. This would explain the localization of TbPEX13.1 to the ER in the absence of glucose, conditions under which de novo biogenesis may be the primary mechanism of organelle formation. Our hypothesis predicts that silencing in glucose-rich media would disrupt the contribution of de novo biogenesis, resulting in the absence of glycosomes in the ER exit area of the cell observed by Kalel et al. [48] but not from other regions of the cell where glycosomes primarily proliferate by fission. TbPEX16 silencing in glucose-poor conditions may result in a more dramatic decrease in the number of glycosomes. Similarly, knockout of TbPEX19 via homologous recombination, cre-lox, or Crispr/CAS in the absence of glucose may result in glycosome-deficient parasites, as mislocalization of glycolytic enzymes is anticipated to be more tolerated in *T*. *brucei* under these conditions [14].

In the future, experiments to characterize peroxin localization and silence peroxins in low-glucose conditions may provide insight into the potential mechanisms of biogenesis and whether they differ with changes in extracellular conditions. Complementing silenced peroxins and restoring glycosome populations would also provide evidence of de novo glycosome formation. While we have only examined the influence of glucose availability, it is likely that other environmental conditions could also influence glycosome biogenesis, such as cell density, pH, osmotic pressure, temperature, and availability of other nutrients. Better understanding of how glycosome populations are maintained and remodeled under different environmental conditions may lead to the identification of new drug targets that could be exploited in the development of therapeutics.

**Key learning points**

1. Glycosomes are considered specialized peroxisomes because they share homologous peroxins involved in proliferation and protein import. Like peroxisomes, they proliferate through growth and division, but a *de novo* mechanism of glycosome biogenesis has yet to be characterized.
2. In yeast and mammalian cells, peroxins have been localized to the ER when *de novo* peroxisome biogenesis is occurring. TbPEX13.1 was recently demonstrated to be localized to the ER in *Trypanosoma brucei*.
3. Silencing of TbPEX16 and TbPEX19, peroxins key for ER-dependent organelle biogenesis, results in a reduction in the total number of glycosomes similar to results obtained in yeast and mammalian studies.
4. Glycosomes are remodeled via autophagy during life cycle differentiation and in response to environmental changes. *De novo* biogenesis of glycosomes could help to restore glycosomes following organelle turnover.
5. These findings suggest that glycosomes proliferate through growth and division of existing organelles and through *de novo* biogenesis from the ER.

**Five key papers in the field**

1. Güther MLS, Urbaniak MD, Tavendale A, Prescott A, Ferguson MAJ. High-confidence glycosome proteome for procyclic form trypanosoma brucei by epitope-tag organelle enrichment and SILAC proteomics. J Proteome Res. 2014;13(6):2796–806.
2. Bauer ST, Mcqueeney KE, Patel T, Morris MT. Localization of a Trypanosome Peroxin to the Endoplasmic Reticulum. 2016;(864):1–9.
3. Kalel VC, Schliebs W, Erdmann R. Identification and functional characterization of Trypanosoma brucei peroxin 16. Biochim Biophys Acta—Mol Cell Res [Internet]. 2015;1853(10):2326–37.
4. Banerjee SK, Kessler PS, Saveria T, Parsons M. Identification of trypanosomatid PEX19: Functional characterization reveals impact on cell growth and glycosome size and number. Mol Biochem Parasitol. 2005;142(1):47–55.
5. Herman M, Pérez-morga D, Schtickzelle N, Michels PAM. Turnover of glycosomes during life-cycle differentiation of *Trypanosoma brucei*. Autophagy. 2008;4(3):294–308.
